# Collaborative Complete Coverage and Path Planning for Multi-Robot Exploration

**DOI:** 10.3390/s21113709

**Published:** 2021-05-26

**Authors:** Huei-Yung Lin, Yi-Chun Huang

**Affiliations:** 1 Department of Electrical Engineering, Advanced Institute of Manufacturing with High-Tech Innovation, National Chung Cheng University, Chiayi 621, Taiwan; 2 Department of Electrical Engineering, National Chung Cheng University, Chiayi 621, Taiwan; yichun@godel.ee.ccu.edu.tw

**Keywords:** mobile robot, multi-robot system, complete coverage, path planning

## Abstract

In mobile robotics research, the exploration of unknown environments has always been an important topic due to its practical uses in consumer and military applications. One specific interest of recent investigation is the field of complete coverage and path planning (CCPP) techniques for mobile robot navigation. In this paper, we present a collaborative CCPP algorithms for single robot and multi-robot systems. The incremental coverage from the robot movement is maximized by evaluating a new cost function. A goal selection function is then designed to facilitate the collaborative exploration for a multi-robot system. By considering the local gains from the individual robots as well as the global gain by the goal selection, the proposed method is able to optimize the overall coverage efficiency. In the experiments, our CCPP algorithms are carried out on various unknown and complex environment maps. The simulation results and performance evaluation demonstrate the effectiveness of the proposed collaborative CCPP technique.

## 1. Introduction

Due to the high demand of industrial applications and the recent advances of IoT (Internet-of-Things)-based appliances, the latest robotics research has covered a wide range of diverse areas [1]. One specific important topic related to environment exploration is the complete coverage and path planning (CCPP) problem for mobile robots. Its main objective is to find a path for the robot such that every location in the workspace can be visited. The techniques are commonly adopted to explore the environment or structure of interest comprehensively for inspection purposes [2]. In the past few decades, CCPP algorithms have attracted much attention in the robotics research community due to their practical applications to cleaning, mining, agriculture and rescue robots, etc. Although technical challenges consist of various aspects of complete coverage and path planning methods, the essential issue lies in the exploration of an unknown environment.

The key factors for exploration include the completeness and time efficiency (and other specific costs) for the environment coverage. In the literature, complete coverage is a classic computational geometry problem and various approaches have been proposed [3]. One class of simple yet robust methods is to utilize the divide and conquer strategy. This approach partitions the entire environment into a number of subregions called cells. Each cell is then covered by a few fundamental simple robot motion paths such as zigzag or spiral [4]. This decomposition is finally assembled to form the full coverage of the space. Since the solutions to the unconstrained complete coverage problem have been extensively investigated, the current developments for robot exploration applications mainly focus on the coverage efficiency [5]. This might consist of the overall execution time for the complete coverage task, the total travel length of the motion trajectory, and the efficiency for energy consumption [6], etc.

In general, CCPP algorithms can be categorized into offline and online approaches [7]. The offline algorithms are designed for situations where the information of the environment is available a prior and the motion path can be planned before executing the exploration task. To deal with the obstacles inside the region of interest in space,  area decomposition methods are commonly adopted. A unified mesh structure is used to represent the entire environment, and a local coverage is carried out on the individual triangular meshes. Alternatively, the Voronoi diagram can be utilized to establish the distributed coverage framework [8]. In an early work, Choset proposed the boustrophedon cellular decomposition, which generalized from trapezoidal decomposition but allowed non-polygonal obstacles [9]. The coverage problem was then reduced to finding the optimal path from the adjacent graph representing the cells. González et al. presented a backtracking spiral algorithm for complete coverage [10]. It can be combined with general grid-based algorithms to improve the performance of structured filling paths [11].

On the other hand, the online algorithms aim to perform the exploration and update the map coverage based on the information collected in the environment. It usually considers the unknown space for exploration, and minimizes the overall cost for complete coverage while maintaining the least revisit areas. In this regard, Liu et al. proposed an algorithm which adopted the random path planning, followed by a comb-like path for local complete coverage [12]. It was successfully applied to the vacuum cleaning robots with low-cost hardware in a small unknown environment. Oh et al. presented a map representation based on triangular cells for cleaning robot navigation [13]. They combined the wall-following and template-based local navigation to achieve the complete coverage. More recently, an online coverage path planning algorithm $\varepsilon^{🟉}$ was proposed for unknown environment exploration [14]. It utilized a so-called exploratory turing machine to store and update the information of explored and unexplored regions at a time-varying basis. The adaptive navigation can avoid the local extrema and is computationally efficient.

In addition to the general constraint on the travel length for coverage, the energy consumption is also an essential issue for the exploration task. It is specifically important for real situations when the mobile robot operates under the battery-powered limitation or the outdoor environment [15]. A complete coverage might require the robot to recharge multiple times for a large environment. The full exploration with an energy cost was first studied by Shnaps and Rimon using a model approximated with the path length [16]. Based on the similar energy-constrained concept, Wei and Isler proposed a constant-factor approximation algorithm [17]. Under the assumption of contour-connected environment, the robot was restricted to parallel motion and tested on the field experiment with an unmanned aerial vehicle.

The learning based approach has been investigated for complete coverage and path planning in recently years. One particular method is the use of reinforcement learning (RL) techniques to deal with the task modeled by the traveler sales problem (TSP). In [18], Le et al. proposed a complete path planning algorithm using a TSP-based RL optimization method for a tiled robot. The reward of the trained RL model was maximized to derive the robot shapes and trajectories. It provided the comparable results obtained from the ant colony optimization approach in terms of energy and time. They have also applied the similar technique on a self-reconfigurable robot with adjustable dimensions [19]. With the emphasis on the tileset energy-aware CCPP, the proposed method had better performance compared to the genetic algorithm (GA). To deal with the CCPP problem in a large complex environment, Kyaw et al. formulated it using TSP and deep reinforcement learning (DRL) [20,21]. The recurrent neural network (RNN) was trained using RL and combined with the cellular decomposition method to iteratively generate the coverage path. Schäfle et al. presented a GA-based method to generate rectilinear paths for complete path planning [22]. The cost function was evaluated using the energy to optimize the path planning with respect to the energy consumption.

**Figure 1:**
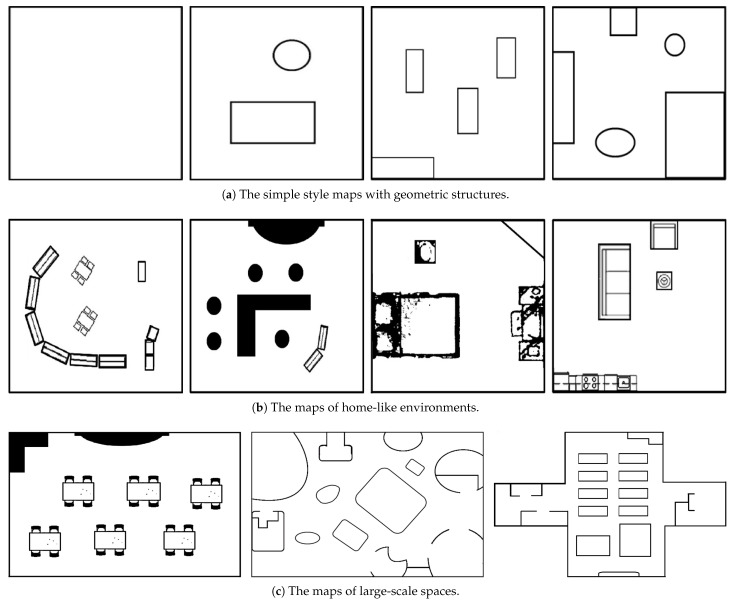
Several typical maps of interest used to evaluate the proposed CCPP algorithms. There are simple style maps with geometric structures, home-like environments, and large-scale spaces.

In this paper, we propose the complete coverage and path planning techniques for the applications of single robot and multi-robot system. A few typical maps of interest are illustrated in Figure 1. Alternatively, the simulation environments using fundamental geometric primitives can be adopted for the construction of various application scenarios [23]. It consists of the maps with simple geometric structures, home-like environments and large-scale spaces. The objective is to reduce the overall computation time for complete coverage by minimizing the repeated region visit via the optimal path planning. For some application scenarios such as finding scattered articles or checking abnormal events in a large-scale environment, it is desired to lower the excessive labor by robotics and automation. This work investigates the collaborative exploration strategy for the full coverage of an unknown space with a team of mobile robots. A new cost function is proposed to evaluate the local exploration gain from the incremental movement. We develop the multi-objective CCPP algorithms and use 2D grid maps for implementation. The experiments carried out on various environments have demonstrated the efficiency of our collaborative CCPP approach for multi-robot system through the incremental exploration minimization.

## 2. Multi-Robot System and Cost Function

In the past few years, the applications using multiple robots have been widely available. Despite the advantages of cooperation and collaboration, the multi-robot research is more challenging due to the complication on the objective definition, information sharing, and task assignment [24]. It is commonly required to have a clear problem statement, and the development for the multi-robot system is then used to improve the efficiency. For the environment exploration task, the existing techniques using multiple robots are relatively limited. The difficulties usually lie on the real-time communication and online decision-making among the mobile robots. To cope with the complete coverage and path planning for multiple agents, one approach is to modify the efficient algorithms studied for a single robot to the multi-robot collaboration.

Most of the early works on multi-robot exploration do not explicitly consider the completeness of the space. Yamauchi proposed a frontier-based method where each robot maintained its own global map [25]. Since the decision about where to explore was made independently, the global optimization was not guaranteed. In [26], Burgard et al. presented a technique to reach the same location by collaborative multi-robot exploration in an unknown environment. The basic idea was to reduce the exploration time based on the cost evaluated by the path length. A greedy algorithm with a goal selection function was adopted in the implementation. This concept later became an important reference for efficient exploration. Xin et al. proposed a distributed motion planning algorithm for the cooperative multi-area coverage (CMAC) task [27]. A multi-point dynamic aggregation (MPDA) model [28] was used to formulate the cooperative motion planning for multiple robots. Nevertheless, their experiments carried out on isolated regions with zigzag motion were fairly straightforward. Senthilkumar and Bharadwaj modified the spanning tree coverage (STC) technique [29] for multi-robot exploration and coverage in an unknown terrain [30]. An extended scheme of multiple STC was used to cover the continuous and bounded region represented by the cellular decomposition. Thus, the complete coverage can be performed in small areas with partially occupied cells and narrow door openings.

Unlike previous approaches, we tackle the CCPP problem starting with the design of a suitable cost function. With the emphasis on the exploration of an unknown environment, the complete coverage is achieved by the cost evaluation and the corresponding motion strategy. Inspired by the work of frontier-based map exploration using particle swarm optimization (PSO) [31], the goal selection and incremental exploration are designed according to the equation

(1) $$x_{i}\left(t+1\right)=x_{i}\left(t\right)+v_{i}\left(t+1\right)$$

where $x_{i}\left(\cdot \right)$ and $v_{i}\left(\cdot \right)$ are the position and movement of the *i*th robot, respectively. In our approach, a guided trajectory is constructed to perform the maximum coverage. The cost function for the heading direction ϕ of the robot is defined by

(2) $$C\left(\phi \right)=\lambda \cdot \left(\rho +\frac{\phi }{\eta }\right)+\bar{\lambda }\cdot \frac{\phi }{\alpha }$$

and practiced on the simulation environment to improve the coverage efficiency.

The cost evaluated by Equation (2) is able to prevent the motion path too close to the obstacles (or walls), and maximize the exploration gain. The parameters in the equation are described as follows. First, ρ is the shortest distance between the robot and the midpoints of a pair of obstacles. It is set as zero if no disjointed obstacles are discovered at the current robot position. Second, the robot heading is denoted by the steering angle ϕ, and the range is between −90 and 90 degrees. Third, the parameter η represents the information related to the obstacles. It is defined by the accumulated obstacle points during the navigation. In the implementation, a laser range scanner is adopted for the environment exploration. It is used to detect the obstacles within a fixed distance and $180^{\circ }$ field-of-view. The sampling rate of laser range scanning is every 5 degrees for the detection of each obstacle point), i.e.,   

(3) $$\eta =\frac{\sum_{j=0}^{t}\sum_{\theta =-90}^{90}\zeta \left(\theta \right)}{\sum_{j=0}^{t}EA_{j}}$$

where $EA_{j}$ is the exploration area from the beginning at time j=0 to the current time *t*, and ζ(θ) indicates the obstacle point at the laser range-finder scanning angle θ. Since we are only interested in the forward motion, the range of θ is between −90 and 90 degrees (with the heading direction at $0^{\circ }$). Fourth, α is the coverage gain of the incremental movement. Finally, λ is a binary number to select either one of the two cost terms in Equation (2) for computation. If there are obstacles scanned between −15 and 15 degrees or no exploration gain, λ is set as 1; Otherwise, λ=0. This is to increase the possibility of covering the unknown space with the maximized area gain. The range setting of −15 to 15 degrees aims to encourage the robot to move forward if there are no obstacles in this scanning range, instead of moving in other directions. These values are determined empirically in our implementation.

When there is no coverage gain, i.e., λ=1, it is required for the robot to move to a new location and continue the exploration. The ideal goal position needs to satisfy the following two criteria.

- The repeated coverage is minimized on the way to the new location.
- The robot will not move the new location before finishing the coverage locally.

Thus, the goal selection function is defined by

(4) τ=max(α,η)

and the procedure is shown in Algorithm 1. In the algorithm, the candidate location is found in the range of −90 to 90 degrees from the mobile robot’s heading angle. It checks the direction which contains less information related to the obstacles and coverage in terms of higher α and η. To make the robot move to the goal position, the Theta* algorithm proposed by Viet et al. is adopted [32]. It is originally designed for a team of robots to cover an unknown space using the Boustrophedon method. When the goal position is reached, the multi-target Theta* algorithm is utilized to trace the record of backtracking points. That is, the path derived from the multi-target Theta* algorithm is recorded and adopted for our path planning. The one with the shortest traceback path is then set as the next starting position for the continuing exploration.
**Algorithm 1** Goal Selection Algorithm**Require:** The robot position**Ensure:** The robot position 1:If λ==0, then 2:   Collect the sensor information from $-90^{\circ }$ to $90^{\circ }$, find the location with the most *coverage free*. 3:   Set a node for every five degrees. 4:Combine the information from Steps 2 and 3 to find the location with max(α,η). 5:Move to the target location. 6:If the explored location to the *unknown* location, then 7:   Use the Theta* algorithm to reach the target location. 8:Else 9:    Find the information for coverage according to cost function. 10:  Go to Step 6.

## 3. Complete Coverage and Path Planning

Based on the cost function design presented in Section 2, the complete coverage and path planning algorithms are proposed for single robot and multi-robot system. The grid maps are constructed by cells with binary values, and the coverage is performed step by step under the robot movement. A simulation interface is developed for the verification of our algorithms and the demonstration of the coverage results.

Similar to the technique proposed by Pereira et al. to cover the unknown environment [33], our exploration method adopts the individual decision rules for the robots, and shares the same environment map via the online communication to minimize the coverage overlap. The frontier-based exploration algorithm is used with the emphasis on the multi-robot coordination and the strategy for new position selection. The main objective is to constrain the steering angle change of the mobile robot, and move to the next unknown area for exploration. Alternatively, we can also consider the A* or D* algorithms for trajectory optimization [34,35]. In the collaborative exploration, each robot will broadcast its location and exploration history on the map. A consistent global map is updated among the multiple robots for future path planning. Algorithm 2 shows the basic multi-robot exploration algorithm. In the algorithm, the global map is first created with a 2-dimensional array to store the status values for all cells. This array is maintained and updated for each movement of the individual robots.
**Algorithm 2** Basic Multi-Robot Exploration Algorithm**Require:** A team of mobile robots**Ensure:** Explored map created with a 2-D array 1:**repeat** 2:    Update the 2-D map, read data from the sensor. 3:    Detect the obstacle locations. 4:    Rotate the robot to keep the largest distance, and follow the wall to explore. 5:    Send the current 2-D map array to other robots for update. 6:    Confirm the exploration information. 7:    Update the global map. 8:**until** All information {obstacle, coverage free} filled.

To make it easier for the algorithm verification in the simulation environment, it is assumed the real-time communication among multiple robots is available. Thus, Steps 5 and 6 in Algorithm 2 will not be affected. In an advanced collaborative exploration algorithm, the costs of multiple robots are sorted. It is used to increase the efficiency of the multi-robot system by the arrangement of individual robots. Algorithm 3 outlines the details of the improved multi-robot exploration strategy. In this algorithm, each mobile robot checks the status and updates the global map through *RobotHeading*. It provides the information about which specific area is best suited to be explored by a certain robot. To achieve the optimal movement of a multi-robot system, the costs of all robots are sorted and the one with the smallest value is used to cover an unknown region. The cost functions of other robots are then evaluated again for the exploration of the next area.

One of our main objectives is to completely cover a large-scale unknown space using multiple robots. There could be possible coverage issues such as the environment containing dense obstacles and many scattered unknown areas. To ensure the robot to find the best target position with the efficient path planning, several additional features are adopted. As given in Algorithm 4, when a mobile robot with the smallest cost is found in the exploration, other robots will not choose to cover the same unknown area. The coverage gain α is checked sequentially for the individual robot to derive the best exploration area.

It should be noted that, although the algorithms are presented for multi-robot complete coverage and path planning task, we do not provide a theory or theoretical analysis on this problem. There might not be an optimal solution for the diverse and complicated environment maps. The intuition behind the design of our cost function is to maximize the exploration gain for each robot movement while reducing the possible coverage overlap among multiple robots. For the collaborative exploration among multiple robots, we evaluate the local gain in the neighborhood of individual robots, and provide the global uncovered areas for all robots as a guidance to discovery. The algorithms are guaranteed to terminate when all region borders (including obstacles) are observed, which indicates the complete coverage by definition.
**Algorithm 3** Improved Multi-Robot Exploration Algorithm1:If *all data* in the exploration is *coverage free*, then2:   Check *RobotHeading* and the global map.3:Else4:   If the *unknown area* obtains *coverage free* and *obstacle data*, then5:      Sort the costs of all robots; high η and low rotation angle receive a higher priority.6:   Else7:      The robot with the shortest distance is to explore the area.8:   Explore with the largest α.9:If two robot repeat the same area for exploration, then10:   Go to Step 4.11:Else12:   Go to Step 1.
**Algorithm 4** Advanced Multi-Robot Exploration Algorithm**Require:** A team of robots**Ensure:** The exploration map 1:**repeat** 2:    Initialize the global map. 3:    Move by the cost, update the map, and label with $\mu_{1}$ if not filled from the movement. 4:    Check *RobotHead* if two *obstacles* having *coverage free*. 5:    Yes 6:       Rotate with the highest η, label the rotated target as $\mu_{2}$, and label the other side as $\mu_{3}$. 7:       Apply the Theta* algorithm to complete the $\mu_{1}$ exploration for the closet *unknown* area. 8:    No 9:       Go to Step 3. 10:    Check the robots for the same coverage area. 11:**until** All information {obstacle, coverage free} filled.

## 4. Implementation and Experiments

**Figure 2:**
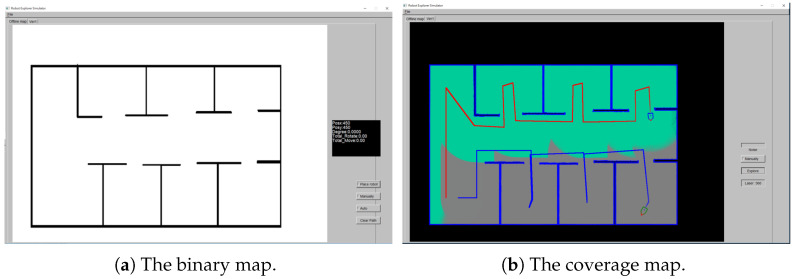
The application interface of the complete coverage and path planning simulation environment used for the multi-robot exploration.

In our implementation and experiments, the graphical user interface for the simulation environment is developed. It is constructed using FLTK as the cross-platform SDK, OpenGL for graphics drawing, and OpenCV for the map image reading. The hardware system for the implementation is a PC with Intel Core i7-4700 CPU @ 3.6 GHz and 8 GB RAM, running on Microsoft Windows 10 64 bits OS. As illustrated in Figure 2, the application interface provides a 1300×900 window size to accommodate a large-scale environment map. An example of the binary image map and the complete coverage result are illustrated in Figure 2a,b, respectively. In the simulation environment, the field-of-view (FOV) of the laser ranger scanner can be set as $120^{\circ }$, $180^{\circ }$ or $360^{\circ }$. This is used to simulate the application scenarios of the real environments which adopt a high-precision optical scanning device [36,37]. There are eight directions for the robot movement, namely left, right, front, rear, front left, front right, rear left and rear right, and used as the parameter *RobotHead* in the algorithm. The direction of *RobotHead* is determined according to the cost evaluation. For the non-omnidirectional motion setting, the angle is derived in the range of −90 to 90 degrees. It is then assigned to one of the five orientations at $-90^{\circ }$, $-45^{\circ }$, $0^{\circ }$, $45^{\circ }$ and $90^{\circ }$ for movement.

**Figure 3:**
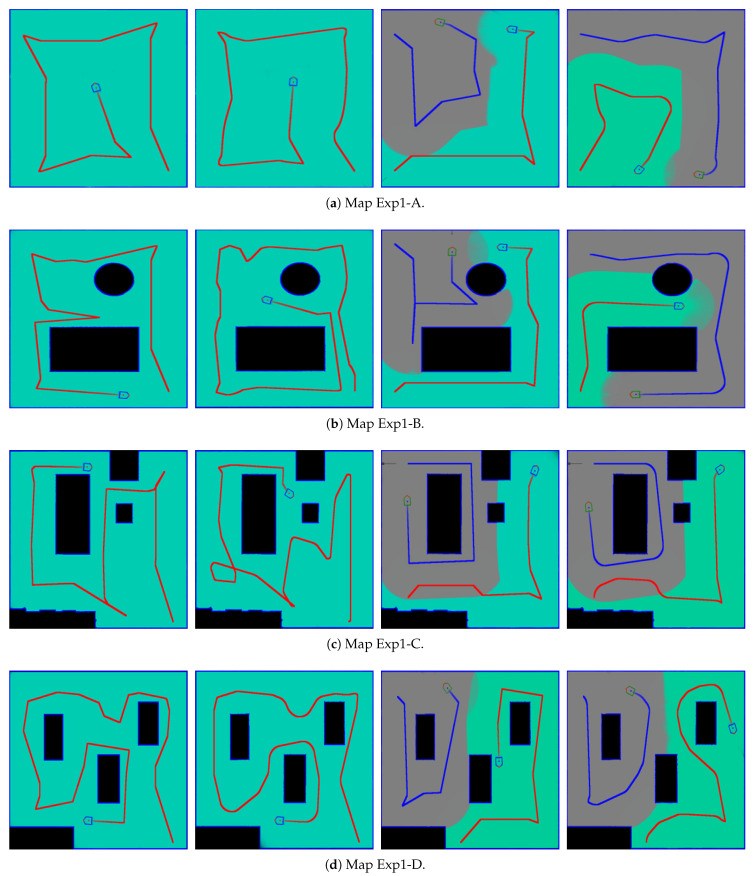
The results of complete coverage and motion path for the two cases: full stop and slow down before making turns. From the left to the right: single robot with full stop, single robot with slow down, multiple robots with full stop, multiple robots with slow down. Observe that the trajectories can be fairly diverse due to the different motion control methods.

Three types of maps are created to evaluate the proposed CCPP algorithms for the exploration using single and multiple robots. The map design consists of *simple geometric structures*, *home-like environments* and *large-scale space*, as shown in Figure 1. In the first experiment, we investigate the coverage efficiency associated with the turning and forward motion of the robot. The comparison is carried out on simple map layouts using Algorithm 3. Table 1 tabulates the comparison of the exploration time when the robot *performs a full stop* or *slows down* before making turns. Figure 3 shows the results of complete coverage and motion path for these two cases. The regions explored by the robots with the visible range are indicated using the green and gray colors. The exploration process terminates if all unveiled areas are enclosed by the boundaries. In the evaluation, the results illustrate that the performance is not necessarily improved by slowing down before making turns. Thus, the further simulation experiments will be carried out with a full stop before the robot heading direction change.

**Figure 4:**
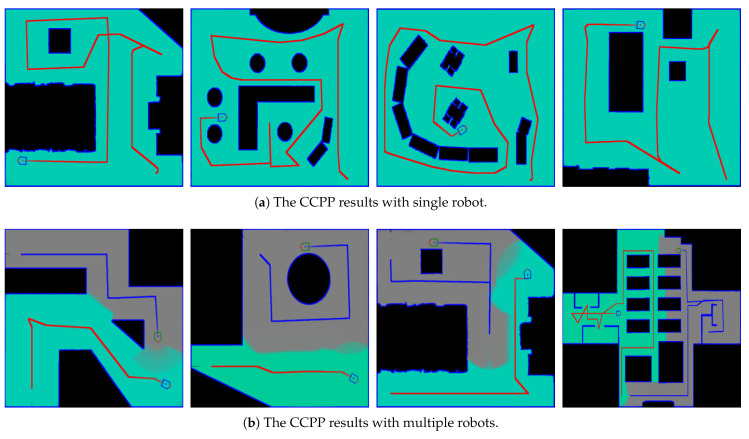
The exploration results with single robot and multi-robot system.

**Figure 5:**
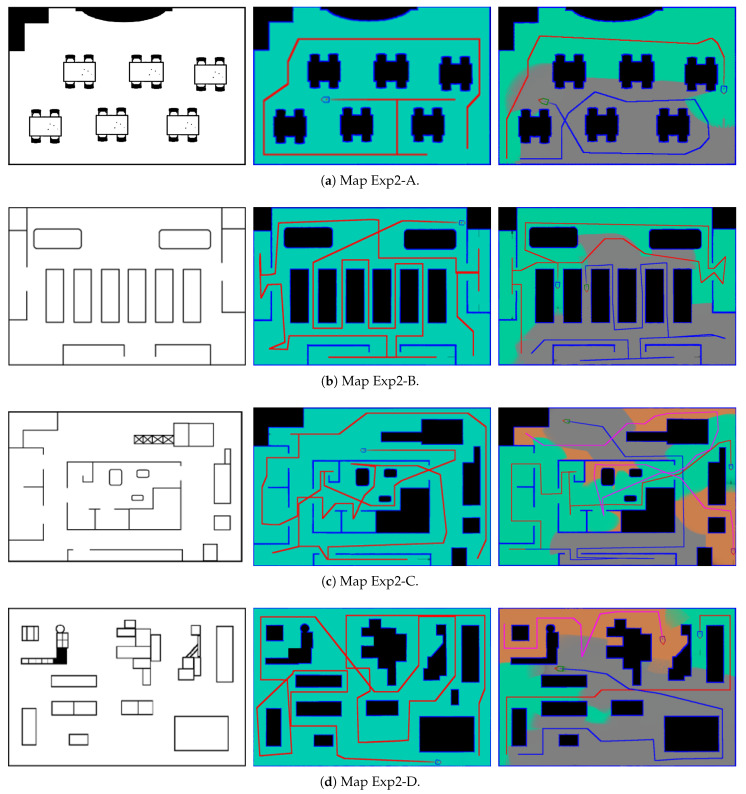
The efficiency of our algorithms is evaluated using four different maps. The left to the right: the original maps, the coverage results of single robot, the coverage results of multiple robots.

**Figure 6:**
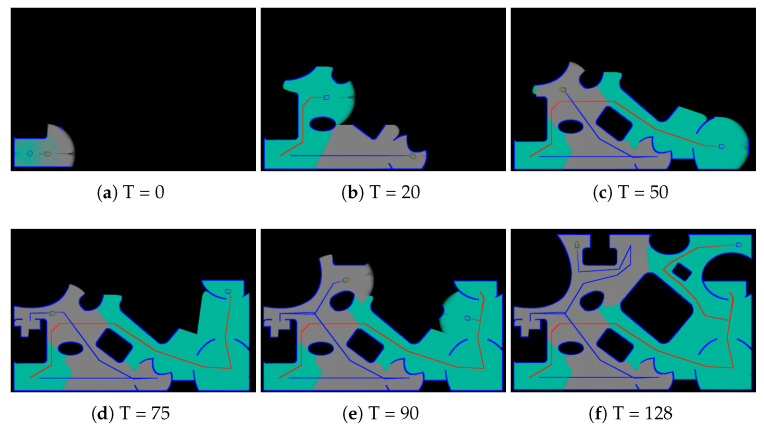
An example of the exploration process using the proposed multi-robot CCPP algorithm. The figures indicate that the high coverage gain is an important criterion for the robot movement.

**Figure 7:**
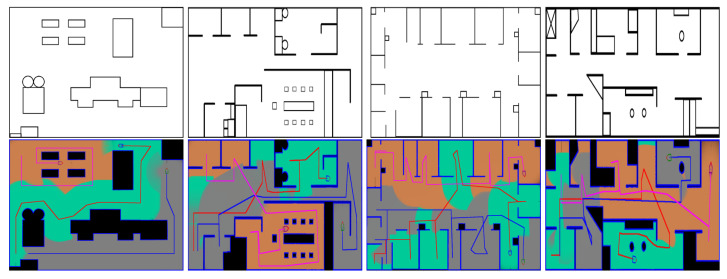
Some additional complete coverage results of the multi-robot system with more complex environment maps. The experiments are carried out using the proposed algorithm with three robots.

In the second experiment, we demonstrate the complete coverage and path planning results using the proposed collaborative CCPP algorithms. Figure 4a,b shows the exploration results with single robot and multi-robot system, respectively. The efficiency of our algorithms is evaluated using four different maps, and the complete coverage results are shown in Figure 5. Table 2 tabulates the exploration time for single robot and multi-robot system on the four maps. The evaluation shows the improvement when multiple robots are deployed for the complete coverage. An example of the exploration process using the multi-robot CCPP algorithm is illustrated in Figure 6. The figures indicate that the high coverage gain is an important criterion for the robot movement. Several additional complete coverage results using multiple robots for the large environment and complex workplace are shown in Figure 7.

**Figure 8:**
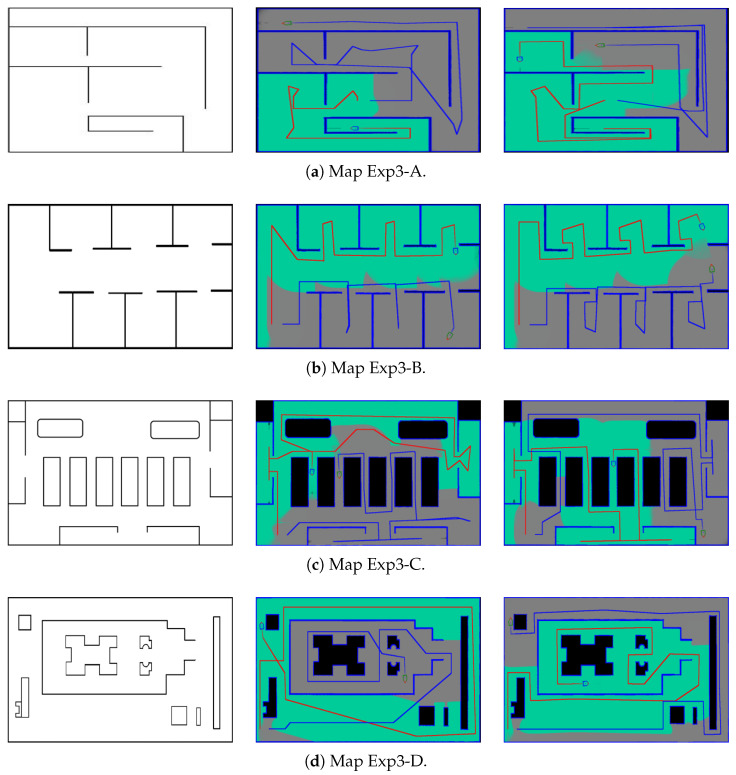
The complete coverage results of the multi-robot exploration using our technique and the algorithm proposed by Pereira et al. [33]. From the left to the right: the original maps, the coverage results using our technique, the coverage results using Pereira’s algorithm.

Finally, our multi-robot exploration method is compared with the technique proposed by Pereira et al. [33]. The algorithm adopts a leading edge based method and combines with the coordination of multiple robots using topological maps for full coverage. Figure 8 shows the complete coverage results of the two methods. The exploration times are tabulated in Table 3. In general, Pereira’s technique provides lower coverage efficiency in small rooms, as the trajectories shown in Figure 8a,b. Thus, the reduction of the processing time using our algorithm is more significant (up to 37%). Moreover, we are able to provide better coverage results for all cases. In the proposed algorithms, the trajectories depend only the layout and obstacles in the map. Since it is assumed that no dynamic objects in the environment, our techniques are constantly stable in the experiments.

In our experimental results, it is first demonstrated that the motion control behavior, i.e., *performing a full stop or slowing down before making turns* do not affect the coverage efficiency in a noticeable way. Thus, it is preferable not to consider the complicated motion acceleration for robot control. Second, the collaborative multi-robot algorithm implementation for complete coverage indeed provides much less exploration time. It is able to minimize the overlapping coverage areas to reduce the cost on multiple visits from different robots. In the proposed techniques, the coverage efficiency is greatly improved, specifically for the maps of large-scale environments.

## 5. Conclusions

In mobile robotics research, one important application is to explore the unknown environments completely and efficiently. In this work, we present the complete coverage and path planning techniques for single robot and multi-robot system. A cost function evaluated based on maximizing the coverage gain is proposed. We design a goal selection function to facilitate the collaborative exploration using multiple robots. By simultaneously considering the local gains from the individual robots and the global optimization among the robots, the overall coverage efficiency is improved. Our proposed algorithms have been tested on various unknown and complex environment maps. The simulation results and performance comparison with the existing approach have demonstrated the effectiveness of the proposed technique. In future work, we aim for our development to focus on the issues of limited or delayed communication among the robots. This is an important and practical problem encountered in real-world multi-robot applications.

## Figures and Tables

**Table 1:** The comparison of the exploration time when the robot performs a full stop or slows down before making turns.

	Single Robot			Multiple Robots		
	Full Stop	Slow Down	Improvement	Full Stop	Slow Down	Improvement
Map Exp1-A	120.64 s	116.47 s	3.3%	47.32 s	54.89 s	−1.6%
Map Exp1-B	118.51 s	121.06 s	−2.1%	54.07 s	47.00 s	13.1%
Map Exp1-C	106.19 s	108.43 s	−2.1%	53.10 s	53.17 s	−0.13%
Map Exp1-D	108.11 s	108.43 s	−0.3%	54.83 s	52.82 s	3.67%

**Table 2:** The exploration time for single robot and multi-robot system on the four maps. The evaluation shows the improvement when multiple robots are deployed for the complete coverage.

	Single Robot	Multi-Robot System
Map Exp2-A	128.52 s	64.84 s
Map Exp2-B	370.35 s	184.50 s
Map Exp2-C	406.69 s	202.38 s
Map Exp2-D	263.37 s	115.96 s

**Table 3:** The performance comparison of our proposed technique with the algorithm presented by Pereira et al. [33].

	The Algorithm in [33]	The Proposed Method	Our Improvement
Map Exp3-A	187.252 s	118.347 s	37.8%
Map Exp3-B	168.816 s	110.924 s	34.3%
Map Exp3-C	203.577 s	184.496 s	9.4%
Map Exp3-D	172.378 s	140.941 s	19.2%
